# SLICE (SMARTS and Logic In ChEmistry): fast generation of molecules using advanced chemical synthesis logic and modern coding style

**DOI:** 10.1186/s13321-025-01119-9

**Published:** 2025-12-09

**Authors:** Stefi Nouleho Ilemo, Victorien Delannée, Olga Grushin, Philip Judson, Hitesh Patel, Marc C. Nicklaus, Nadya I. Tarasova

**Affiliations:** 1 Cancer Innovation Laboratory, Center for Cancer Research, National Cancer Institute, National Institutes of Health, P.O. Box B, Frederick, Maryland 21702 USA; 2 Laboratory of Chemical Biology, Center for Cancer Research, National Cancer Institute, National Institutes of Health, Frederick, Maryland 21702 USA; 3 Present Address: Deep Origin, San Diego, CA USA; 4 Frederick National Laboratory for Cancer Research in the Cancer Innovation Laboratory, Center for Cancer Research, National Cancer Institute, National Institutes of Health, Frederick, Maryland 21702 USA; 5 Heather Lea, Bland Hill, Norwood, Harrogate, HG3 1TE England; 6 Present Address: OpenEye, Cadence Molecular Sciences, Santa Fe, NM USA

**Keywords:** Virtual libraries, Virtual ligand screening, Structure-based drug discovery, Chemical spaces

## Abstract

While virtual libraries of synthetically accessible compounds have exploded in size to many billions, our capacity to extract valuable drug leads from these vast databases remains limited by computational resources. To overcome this, we developed SLICE SMARTS and Logic In ChEmistry), a powerful new tool designed for the agile exploration of massive chemical spaces. SLICE enables the fast, “à la carte” generation of virtual compound libraries through chemist-defined reaction chemistries and readily available building blocks. Its user-friendly, no-code graphical interface, the SLICE Designer, allows chemists to easily define SMARTS patterns, configure atom and bond properties, and establish chemical constraints and logic. The resulting XML files are then fed into the SLICE Engine, which generates diverse virtual libraries from specified building blocks at speeds of 0.6–2.5 million compounds per hour. SLICE provides the agility and performance needed to support efficient lead generation within discovery workflows.

**Supplementary Information:**

The online version contains supplementary material available at 10.1186/s13321-025-01119-9.

## Scientific contribution

We have developed a novel computational framework that enables the rapid, straightforward, and highly targeted generation of custom virtual libraries of synthesizable compounds. By providing prompt and precise access to the most appropriate regions of the chemical universe, our program substantially accelerates the drug discovery process.

**Supplementary Information:**

The online version contains supplementary material available at 10.1186/s13321-025-01119-9.

## Introduction

Virtual screening of synthetically accessible compounds libraries is an increasingly vital step in modern drug discovery [1–6]. This growth is driven by significant advances in protein structure determination, the availability of vast chemical databases, increasing computational power for screening, and breakthroughs in AI. AI’s role extends beyond protein structure prediction to improving scoring functions and optimizing protein structures [7–10]. However, navigating the immense chemical universe—estimated to contain over 10^60^ compounds [11] — to access drug-like molecules remains a formidable challenge. While we have the starting data to generate trillions or more virtual synthesizable molecules in the computer, explicitly enumerating these vast chemical spaces is impractical, if not impossible [12]. Consequently, fragment-based approaches have become widely adopted [13, 14]. These methods enumerate only specific portions of the chemical space, enabling the on-demand generation of virtual combinatorial libraries. In addition, new methods for exploring chemical spaces without explicit enumeration are now available, offering promising avenues for faster discovery [6, 15, 16].

Early foundational work, such as LHASA (Logic and Heuristics Applied to Synthetic Analysis), pioneered computer-aided synthesis [17]. LHASA allowed the encoding of chemical transformations using CHMTRN and PATRAN, a powerful framework but one that was code-intensive and demanded significant expertise to operate effectively [18]. The SLICE project originated from the SAVI (Synthetically Accessible Virtual Inventory) initiative, which aimed to create a comprehensive collection of virtual compounds that could be synthesized easily and cost-effectively [19]. SAVI relied on LHASA transforms, written in CHMTRN/PATRAN, to ensure the generation of only synthetically feasible compounds. However, writing and managing (debugging, improving, and tightening chemically, etc.) these transforms in a complex, text-based language poses significant challenges, especially for those without programming expertise. To address these limitations, we developed a fast, user-friendly platform that allows chemists to define chemical transforms using a graphical interface. The platform consists of two parts. (1) SLICE Designer writes chemical rules for compounds generation; and (2) SLICE Engine conducts virtual synthesis. In SLICE Designer, molecules are drawn with an intuitive tool, and reaction logic is constructed using custom-designed Blockly blocks, eliminating the need for traditional programming. SLICE Engine uses the transform’s logic created by SLICE Designer, plus files with starting blocks for reactants as an input. It applies the logic, processes the reaction, and automatically generates the corresponding product compounds. SLICE thus not only enables the definition of reaction rules and logic constraints but also facilitates the accessible and efficient production of virtual compound libraries. Existing cheminformatics tools like RDKit [20], OpenEye [21] KNIME [22], Indigo [23], ChemAxon’s Marvin/JChem [24], and NextMove Software’s Arthor [25] provide valuable capabilities but often necessitate programming knowledge, complex workflows, or commercial licenses. In contrast, SLICE modernizes the core concept of LHASA transforms by offering a graphical, no-code interface. This design provides the speed and compound-generation capabilities essential for today’s demanding discovery workflows. SLICE is primarily designed for chemists and cheminformaticians who need to easily encode chemical reactions for molecule generation. The current version of SLICE Designer allows users to: generate SMARTS patterns by drawing and configuring each atom and bond; store critical information about the chemistry, such as reaction names and conditions; and define specific chemical constraints. Based on this user-supplied information, a SLICE file is generated, ready for immediate use by the SLICE Engine for high-throughput molecule generation. In this paper, we present the motivation, architecture, functionality, performance benchmarks, and comparisons of SLICE. We demonstrate that our solution not only simplifies the authoring of chemical transforms and logic but also enables fast and scalable compound generation for both research and industrial applications.

### Implementation

SLICE is based on expert knowledge used to define rules for the selection of synthetic blocks and generation of products. It is intended to be used by chemists or cheminformaticians who have little or no programming experience as it is based on a simple structure inspired by the home automation software: IFTTT (IF This Then That). We will first describe the SLICE language, then the GUI interface.

#### SLICE as a language

SLICE is an XML-like language, which contains information about transform properties, transform patterns, and associated logic. A transform is a set of rules containing one or multiple reaction(s) of the same type (such as the Suzuki–Miyaura Cross-Coupling reaction) with their associated properties, SMARTS, and logic.

1. Properties

A basic SLICE file contains the transform identifier, the name of the transform, and its current version. Despite all other properties being optional, it stores a history of all modifications made to the transform. The file contains a reference section, where a user can choose to enter bibliographic information related to the transform. Additionally, there is an option to include metrics such as the yield, the reliability, the reputation, the homoselectivity, the heteroselectivity, the orientational selectivity, the condition flexibility, and the thermodynamics of the reaction, and a comments section.

2. Patterns

To recognize templates in a query molecule, SLICE uses SMARTS [26]. SMARTS is a powerful language used to describe substructural patterns in molecules. SMARTS allows the specification of molecular patterns, enabling quick searching and filtering of fragments, functional groups, or specific structural motifs across large chemical libraries containing millions of compounds. In addition to the wide adoption of SMARTS in the cheminformatics community, SMARTS editors have been developed to graphically and interactively edit SMARTS [27]. These tools simplify the SMARTS writing and open it to a non-expert community. All the atom and bond attributes defined in the Daylight Software are implemented in SLICE [26]. Additionally, SLICE implements a few SMARTS extensions, which brought new functionalities to the SMARTS language. For instance, the letter ‘z’ can be used to define the number of heteroatoms. A new attribute referring to the number of electrons has been introduced to capture the complexity of some systems, such as one- and three-electron bonds.

3. Logic

The logic is constructed with predefined blocks, which are combined to define a set of instructions. Block combines conditional statements into a statement with the following basic structure:

if < *subject* >  < *relation* >  < *predicate* >  < *where* > then < *action* > 

 < *subject* > refers to one or more chemical objects related to atoms, bonds, rings, or a molecule (atom 1; alpha to atom 2; bond between atom 2 and atom 3). The keyword *molecule* refers to the whole molecule. < *relation* > defines the relation between the < *subject* > and the < *predicate* > . Forms of the verb “*to be*” are used for qualitative statements (if atom 1 is aromatic), and “to have” for quantitative statements (if atom 1 has at least 1 carbon atom attached to it). < *predicate* > refers to a qualitative property (aromatic, primary center, etc.) or a quantitative property (at least 1 positive charge, at most 2 chlorines, etc.). < *where* > refers to where the chemical objects are in relation to the keying pattern. For instance, *offpath* applies to all chemical objects not included in the keying pattern. < *where* > is optional as its use is unnecessary if the subject is explicitly defined (if atom 1 is aromatic). Finally, < *action* > specifies the action to be triggered if the statement is satisfied. Among the actions, a user can kill a reaction or modify its rating by lowering or raising it. The rating is an assessment of how favorable/unfavorable a specific reaction is. SLICE implements 4 qualitative categories for raising or lowering the rating: slightly, moderately, strongly, and severely. Another action is designating a “ghost molecule.” Many transforms require these to function correctly. Ghost molecules are minor, usually small, fragments that aren’t necessarily “real” molecules. Instead, they’re an artificial construct required to balance a reaction. Multiple statements can be combined using the logical operators “and if”, “or if”, “and”, or “or”. The operators “and if” and “or if” are used to combine multiple statements:

*if atom 1 is the origin of amine1 group*

*or if atom 1 is the origin of amine2 group*

*then lower rating moderately*

*if atom 1 is carbon atom and if atom 1 is aromatic then kill*

Note that the statements presented above can be written on a single line or multiple lines. The operators “and” and “or” are used to combine multiple subjects and/or predicates:

*if atom 1 or atom 2 is the origin of amine1 group then kill*

*if atom 1 is carbon atom and aromatic then kill*

*if atom 1 or atom 2 is carbon atom and aromatic then kill*

In SLICE, a loop is an instruction that iterates one by one over all chemical objects (atom, bond, or group) contained in an object (molecule, set of rings or a set). The structure of a loop is as follows:

foreach < chemical_object >  < *where* > defined as < *variable_name* > in < *Object* > 

 < *where* > is optional and can be left empty. < *Object* > defines a set of chemical objects (i.e. atoms and/or bonds), rings, or the whole molecule. < *where* > indicates where the chemical objects are located relative to the keying pattern. < *variable_name* > is the variable name set by the user. The corresponding variable is initialized to the value taken by the chemical object during each iteration. For instance, in the following example, all offpath carbon atoms are being iterated. If the molecule has 3 offpath carbon atoms (atom 4, atom 5, atom 6), the variable “carbon_atom” will successively be set to atom 4, then atom 5, and, finally, atom 6. The conditional “if” statement is tested at each iteration with the value taken by “carbon_atom”. Thus, at the second iteration, carbon_atom is equal to atom 5 and the aromaticity of atom 5 is tested. At the next iteration, carbon_atom is equal to atom 6, and the aromaticity of atom 6 is tested.

*//iterate over each offpath carbon atom*

*For each carbon atom offpath defined as carbon_atom in molecule {*

*if carbon_atom is aromatic then raise rating slightly*

}

SLICE also implements common functionalities found in most programming languages, such as sets and functions.

#### Graphical user interface “SLICE designer”

**Fig. 1:**
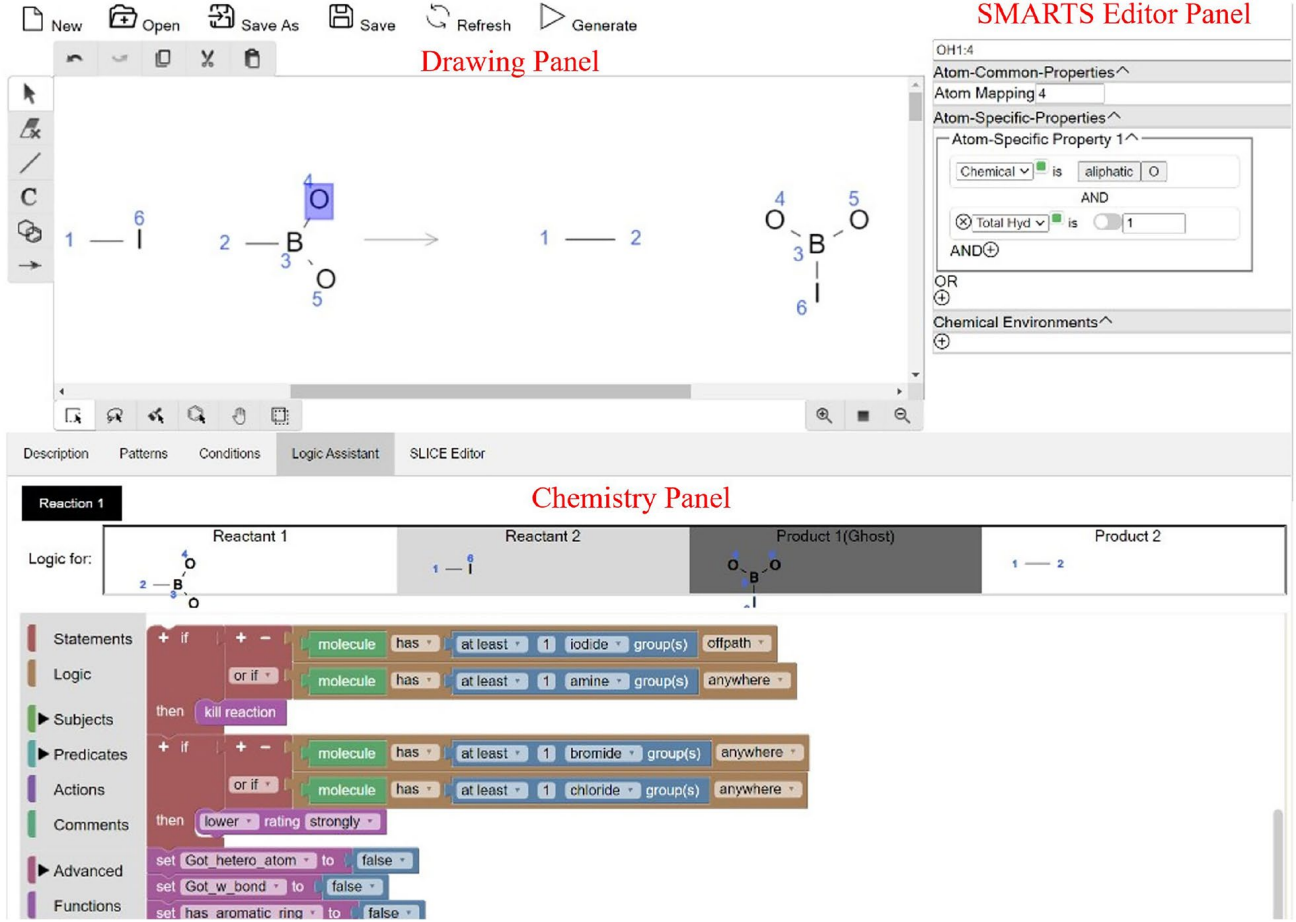
SLICE designer interface with Suzuki–Miyaura cross-coupling (Iodo) transform with logic for reactant 2 open

The SLICE Designer is a graphical user interface (GUI) for creating and editing chemical reaction transforms. It is composed of a menu bar and three main panels: the Drawing Panel, the SMARTS Editor, and the Chemistry Panel (Fig. 1 and summarized in Table 1).

**Table 1:** A guide to the SLICE designer interface panels

Panel	Function
Drawing panel	Use this panel to draw chemical reactions. Molecules are classified based on their position relative to the reaction arrow: reactants are on the left, products are on the right, reagents are above or under the arrow
SMARTS editor panel	This panel allows you to configure individual attributes of each atom and bond. It includes specific properties for both: Atoms: - Atom mapping - Atom properties (Chemical element, Negative Charge, Positive Charge, Connectivity, Degree, Hybridization Number, Total Hydrogen Count, Unsaturation, Periodic Group, Pi electron Count, Ring Bond count, Ring Count, Aliphatic Hetero Substituent Count, Hetero Substituent Count, Valence) Bonds: - Bond properties such as Single, Double, Triple, and Quadruple bonds, as well as Aromaticity, Ring bonds, and Bond Stereochemistry (up, down, etc.)
Chemistry panel	This panel contains five tabs: Description, Pattern, Conditions, Logic Assistant, and Editor
- Chemistry panel: description	Provides general information about the transform including its name, ID, and bibliography
- Chemistry panel: pattern	Automatically displays the SMIRKS of the drawn reaction
- Chemistry panel: conditions	Allows you to enter reaction conditions like temperature and solvent
- Chemistry panel: logic assistant	Allows you to define constraints through interconnected blocks and generates error-free syntax for SLICE logic code
- Chemistry panel: editor	Shows the generated SLICE XML, which is used by the SLICE Engine for generating new molecules

### Menu bar

The menu bar contains standard file management functions such as “New,” “Open,” “Save,” and “Refresh.” The “Save” function creates two files: a.jslice (JSON) file for use with the SLICE Engine and a.slice (XML) file for user readability. The “Generate” function activates the external SLICE Engine to create chemical products based on the open transform file.

### Drawing panel

This panel, based on the kekule.js interface [28], allows users to draw chemical reactions. It includes four toolbars:

- Top Panel: Tools for basic editing like undo, redo, copy, cut, and paste.
- Left Panel: Tools for drawing, including selection, eraser, bond, atom, and ring tools.
- Bottom Left Panel: Displays options specific to the selected tool. For example, selecting the atom tool shows options for creating single atoms or changing atom types.
- Bottom Right Panel: Contains zooming tools.

Molecules are classified as reactants, products, or reagents based on their position relative to the reaction arrow.

### SMARTS editor

This panel allows users to configure the attributes of individual atoms and bonds. When an atom or bond is selected in the Drawing Panel, its corresponding SMARTS Editor is shown. Users can add new attributes with AND + (for complementary properties) or OR + (for independent attributes). The panel includes drop-down menus for selecting attributes, a switch to negate an attribute, and additional options for chemical elements (like aromaticity and a periodic table selector) and quantitative attributes (such as hydrogen count, which can be specified as a fixed value or an interval).

### Chemistry panel

The Chemistry Panel has five tabs that provide optional, but helpful, metadata and logic for the transform.

- Description Tab: For general information like the transform’s purpose, history, bibliography, and notes.
- Pattern Tab: Displays the SMIRKS (SMiles Reaction Specification) of the drawn reaction and the SMARTS of the molecules.
- Condition Tab: Allows entry of general reaction conditions.
- Logic Assistant Tab: A unique feature that helps users build complex constraints using a drag-and-drop block interface. Users select from categories like Statements, Logic, Subjects, Predicates, and Actions to build error-free logic for their reaction. The assistant automatically handles syntax details like variable renaming.
- Editor Tab: Shows the generated SLICE XML code for visualization and verification.

### Generating products

To generate products, the user must open a transform file and select a file of building blocks. The “Generate molecules” button uses these files to create products, which are saved as a Structural Data File (SDF) and/or a.csv file. Users can choose to hide ghost molecules, kill low-rated molecules, or print only InChIKeys to save space.

#### SLICE engine

SLICE Engine is powered by ANTLR (ANother Tool for Language Recognition), which is a parser generator for reading, processing, executing, or translating structured text or binary files [23]. ANTLR uses a grammar file containing a set of rules defining the structure of the language. For instance, the rule ‘capitalized word’ points to a chain of one or more uppercase letters and can be written as ‘capitalized Word: [A-Z] + ;’. The grammar is then automatically converted into a parse tree by ANTLR, which generates the codes that can walk the tree. To offer extended compatibility, the grammar file for SLICE is compatible with both ANTLR versions 3 and 4. This allows the generation of parsing functions in multiple programming languages like C, C +  + , C#, Java, JavaScript, Objective-C, Perl, Python, and Ruby. In the context of SLICE, we chose Java to be able to use the open-source cheminformatics library CDK (Chemistry Development Kit) [29]. CDK is the cheminformatics core of SLICE. It parses the query molecule(s)/reactions, manages the SMARTS patterns, and interprets SLICE logic. CDK will first match a SMARTS pattern with a query molecule. If the pattern matches, the logic applied to the molecule is then checked. The logic is coded by a set of functions in Java that use CDK to reason on a molecule. For instance, the instruction “if atom 1 has one hydrogen” calls a function to select atom 1, another one to get the number of hydrogens, and the last one to evaluate the relation (“has” in this example) and returns a binary answer (true/false).

## Results and discussion

To assess SLICE Designer’s performance, we used a set of transforms previously utilized in the SAVI-2020 project [5] but now applied to a larger building block set provided by Enamine, which included about 284,000 compounds. This ensured a diverse range of chemical structures for testing. This section aims to compare the computation time and the number of products generated by SLICE versus SAVI based on the same 284k building blocks. For this comparison, we selected four specific transforms with rich chemical context annotation including functional group data and scoring systems (as programmed in CHMTRN/PATRAN): 6005 (Suzuki–Miyaura Cross-Coupling (Iodo)), 2875 (Copper[I]-catalyzed azide-alkyne cycloaddition), 2201 (Fused Arylpyridines via o-Aminocarbonyls), and 7009 (Acylsulfonamide from Sulfonamide and Carboxylic Acid). Table 2 outlines the initial runtime and output data for SAVI and SLICE, evaluated across the above four transforms. For each transform, the table shows the number of compatible reactants for both reactant #1 and reactant #2, the number of products after application of the reaction logic, and the total computation time in minutes. Presenting the data side by side provides an overview of each system’s performance in terms of scale and speed. The building block count for reactant #1 and reactant #2 was done based on pattern matching prior to the logic check. The reactant and the product counts may be different for SAVI vs. SLICE due to differences in processing and/or reaction patterns. For both SAVI and SLICE the number of products is the total number of products with a positive scoring.

**Table 2:** Comparison of computation time and number of products in SAVI and SLICE

Transforms	SAVI				SLICE			
ID	#reactant 1	#reactant 2	#products	Time (min)	#reactant 1	#reactant 2	#products	Time (min)
6005	1,218	4022	4,839,627	5519	1226	4221	1,028,513	92
2875	2146	5033	8,602,009	13,449	1939	4078	7,755,571	238
2201	33,668	320	1,190,253	16,038	42,812	316	1,769,041	128
7009	2793	55,408	84,849,166	204,859	3014	56,345	91,265,658	11,305

The number of undesirable compounds produced is highly dependent on the strictness of the transform logic written by a chemist. For instance, the SLICE version of transform 6005, a Suzuki–Miyaura coupling with iodides, is more restrictive than the CHMTRN version. SLICE completely disallows reactions with off-path iodine or any off-path amine, whereas CHMTRN has no such restrictions. Additionally, SLICE lowers the reaction rating for off-path chlorine, bromine, and electron-withdrawing groups on carbocyclic rings in reactant 1, a limitation absent in CHMTRN. In another example, transform 2875, an azide-alkyne cyclization, the SLICE version limits alkynes to terminal acetylenes, while the CHMTRN version does not. This difference results in a significantly higher number of matching reactants and, consequently, more products with the CHMTRN version. Finally, with transform 2201, a synthesis of fused arylpyridines via o-aminocarbonyls, the CHMTRN version includes restrictions on bulky groups adjacent to the reacting carbonyl in reactant 1. The tested SLICE version, however, does not impose these restrictions.

To meaningfully compare CHMTRN and SLICE across transformations with highly variable product counts, we introduced the normalized metric ‘time per million products’. This metric offers a clearer view of computational efficiency by accounting for workload size, addressing the misleading nature of comparing raw runtimes alone, especially when some transformations yield significantly more products. By normalizing performance, we found that SLICE consistently outperforms SAVI, requiring dramatically less time to generate the same number of products, often by an order of magnitude. This demonstrates SLICE’s ability to scale efficiently and perform consistently across diverse transformation tasks. Table 3 presents the normalized performance for four transforms.

**Table 3:** Normalized time per million products for SAVI and SLICE across transforms

Transform ID	SAVI products	SAVI time (min)	SAVI time/million (min)	SLICE products	SLICE time (min)	SLICE time/million (min)	Speed-up ratio
6005	4,839,627	5519	1140.4	1,028,513	92	89.4	12.8
2875	8,602,009	13,449	1563.5	7,755,571	238	30.7	50.9
2201	1,190,253	16,038	13,470.4	1,769,041	128	72.3	186.3
7009	84,849,166	204,859	2413.4	91,265,658	11,305	123.8	19.5

SLICE shows a substantially lower time per million products compared to SAVI, emphasizing its efficiency, especially in smaller-scale transform like 2201. Even though it is difficult to do a fully quantitative comparison between the SAVI and SLICE speeds, given the different programming/scripting languages, workflows, and algorithms used in the two approaches, it is clear that one can expect a speed-up of at least an order of magnitude when going from SAVI to SLICE for the same chemistries.

### Generation of targeted libraries with SLICE

The high-speed generation of libraries by SLICE renders it particularly valuable for fragment-based ligand discovery. Upon identifying a synthetic building block that exhibits favorable binding within a target’s sub-pocket, one can readily generate a focused library utilizing extensive databases of blocks and a variety of chemical transformations. To evaluate SLICE’s performance in generating targeted libraries, we focused on inhibitors of the STAT3 N-terminal domain. (E)-3-(4-hydroxybenzylidene)-2,3-dihydro-1H-cyclopenta[b]quinoline-9-carboxylic acid served as the initial block, owing to its prior identification as a common motif in inhibitors discovered via virtual screening [30]:
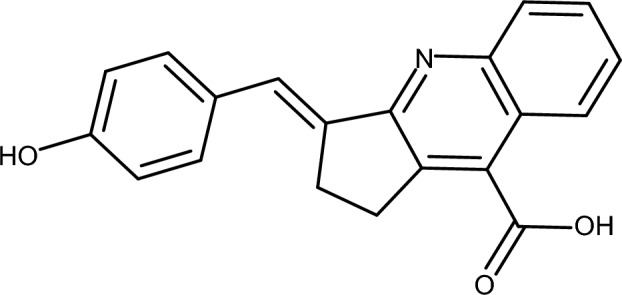


We then employed 1.415 million building blocks from Enamine as a second reagent in two transforms: 7009 (Acylsulfonamide from Sulfonamide and Carboxylic Acid) and 7038 (Amides from Amines and Carboxylic Acids). This resulted in the generation of 11,907 and 144,059 compounds, respectively. Subsequent docking into the STAT3 N-domain structure led to the identification of 18,231 compounds exhibiting improved docking scores relative to the starting lead, ST3-9. Table 4 shows ST3-9 as well as illustrative examples of compounds with significantly enhanced scores, demonstrating SLICE’s capability for accelerated lead optimization.

**Table 4:**
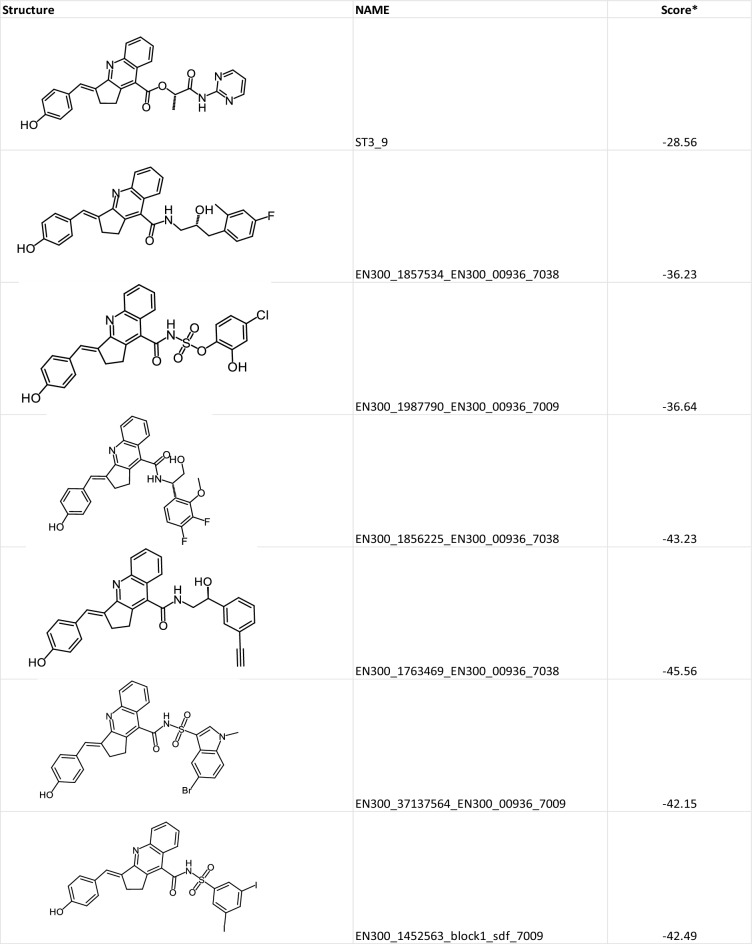
Examples of potential STAT3 N-domain inhibitors with improved scores generated by SLICE

To create diverse screening libraries, it is crucial to utilize a variety of chemistries that can generate different molecular scaffolds and building blocks. The number of commercially available building blocks and new synthetic methods is growing rapidly. The speed of SLICE allows users to capitalize on these advancements by easily incorporating novel chemistries and building block sets, thereby enhancing the diversity of the generated libraries. This offers significant advantages in discovering compounds for challenging targets. Currently, many giga-sized libraries are prefiltered for drug-like properties, often with a molecular weight cut off at 500 Da. However, many clinically used compounds that target “non-druggable” proteins and protein–protein interactions are significantly larger. Therefore, hits from these prefiltered libraries often need to be “grown” to successfully modulate their targets [31]. SLICE can overcome this limitation by generating custom libraries using three and four-step synthesis, which facilitates the creation of compounds tailored for even the most challenging targets.

- Docking score is physics-based score determined using ICM-Pro software as previously described [30]. The lower the score the better the predicted interaction.

## Conclusion

SLICE is a powerful new tool for the rapid, “à la carte” generation of virtual, synthetically feasible compound libraries. While its primary function is molecule generation, the highly adaptable SLICE Designer interface can also be used for other purposes, such as SMARTS editing. The ability for users to view, save, and reload SMARTS patterns makes it a versatile tool for medicinal chemists. Our work demonstrates that SLICE is a significant advancement in navigating the immense landscapes of modern virtual compound libraries. By providing a chemist-friendly, no-code graphical interface for defining complex chemical transformations and building blocks, SLICE streamlines the creation of highly targeted virtual libraries. This platform allows for the programming of novel chemistries as they become available, thereby continuously expanding the accessible chemical space. A critical feature is the SLICE Engine’s ability to generate compounds at a rate exceeding one million per hour, which provides the computational throughput necessary to effectively mine these vast chemical spaces. Ultimately, the combined speed and flexibility of SLICE represent a significant step toward overcoming current computational limitations in drug discovery. This fosters more efficient and successful lead identification. Future improvements will further expand the functionality and accessibility of SLICE. We plan to add support for importing and exporting reaction definitions in.rxn format, complementing the existing utilization of SMIRKS and SLICE formats by the software. While the current multi-processing capabilities utilize all available cores on a single machine, a key future development will be native cloud-based distributed computing support. We are also exploring the integration of property prediction models (e.g., ADME/toxicity) and the inclusion of built-in filters for properties such as drug-likeness.

## Supplementary Information

Supplementary material 1.

## Data Availability

All data and materials discussed in the Results and Discussion section are openly and freely accessible at: https://github.com/tarasovan/SLICE-public/tree/main/examples. This folder contains the following: —**BBS\_Enamine\_284k.tsv**: A dataset of ~ 284,000 Enamine Building Blocks (BBS).—**block1.tsv**: The target compound used for Transform (TF) 7009.—**Four Transforms (TF 2201, TF 2875, TF 6005, and TF 7009)**: For each transform “XXXX,” you’ll find: —TF_XXXX_Vj.jslice: A file required to open the transform in SLICE Designer or run it in the SLICE engine. —TF_XXXX_Vj.slice: A user-readable file detailing the chemistry of the transform. —XXXX_set_BBS_20.txt: A small set of 20 compatible building block reactants.—**script_target_compound.sh**: A script file for generating molecules for a target compound, including the command-line instructions for execution.

## Declarations

### Availability and requirements

Project name: SLICE Engine. Project home page: https://github.com/tarasovan/SLICE-public/tree/main/slice-engine. Operating system: Platform-independent. Programming language: Java. Other requirements: No other requirements are needed beyond a Java Runtime Environment (JRE 8 or higher). Dependencies: Apache Commons (CLI/IO/Math/Lang), Guava, BEAM, JNI-InChI, JAMA, Vecmath, ANTLR 4, univocity-parsers, jopt-simple, Gson. License: MIT license. Any restriction to use by non-academics: none.
