# Supplementary material for: SLICE (SMARTS and Logic In ChEmistry): fast generation of molecules using advanced chemical synthesis logic and modern coding style
Source: J Cheminform. 2025 Dec 9;18:7. doi: 10.1186/s13321-025-01119-9 (PMC12802268; doi:10.1186/s13321-025-01119-9)

**Supplementary SLICE Designer User Guide.**

SLICE (SMARTS and Logic In ChEmistry): Fast
generation of molecules using advanced chemical
synthesis logic and modern coding style

Stefi Nouleho Ilemo^1^, Victorien Delannée^2, 3^, Olga Grushin^4^, Philip Judson^5^, Hitesh Patel^2, 6^, Marc C. Nicklaus^2^, and Nadya I. Tarasova^1^✉️

^1^Cancer Innovation Laboratory, Center for Cancer Research, National Cancer Institute, National Institutes of Health, Frederick Maryland 21702, USA

^2^Laboratory of Chemical Biology, Center for Cancer Research, National Cancer Institute, National Institutes of Health, Frederick Maryland 21702, USA

^3^Present address: Deep Origin, San Diego, CA

^4^ Frederick National Laboratory for Cancer Research in the Cancer Innovation Laboratory, Center for Cancer Research, National Cancer Institute, National Institutes of Health, Frederick Maryland 21702, USA

^5^Heather Lea, Bland Hill, Norwood, Harrogate, England

^6^Present address: OpenEye, Cadence Molecular Sciences, Santa Fe, NM

This manual explains how to write SLICE file and generate products using SLICE Designer interface. For this purpose, we will use the CHMTRN transform 7009 used in SAVI 2020 and described [here](https://cactus.nci.nih.gov/download/savi_download_transformwise/savi_2020_transform_wise_pages/7009.html).


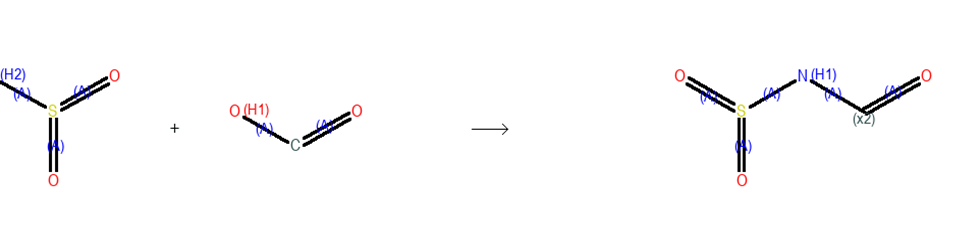


**Step 1: Draw the Reaction**

Use the Drawing Panel to sketch your chemical reaction. Ensure that reactants are on the left side of the reaction arrow and products are on the right. If you are using reagents, place them above the arrow.


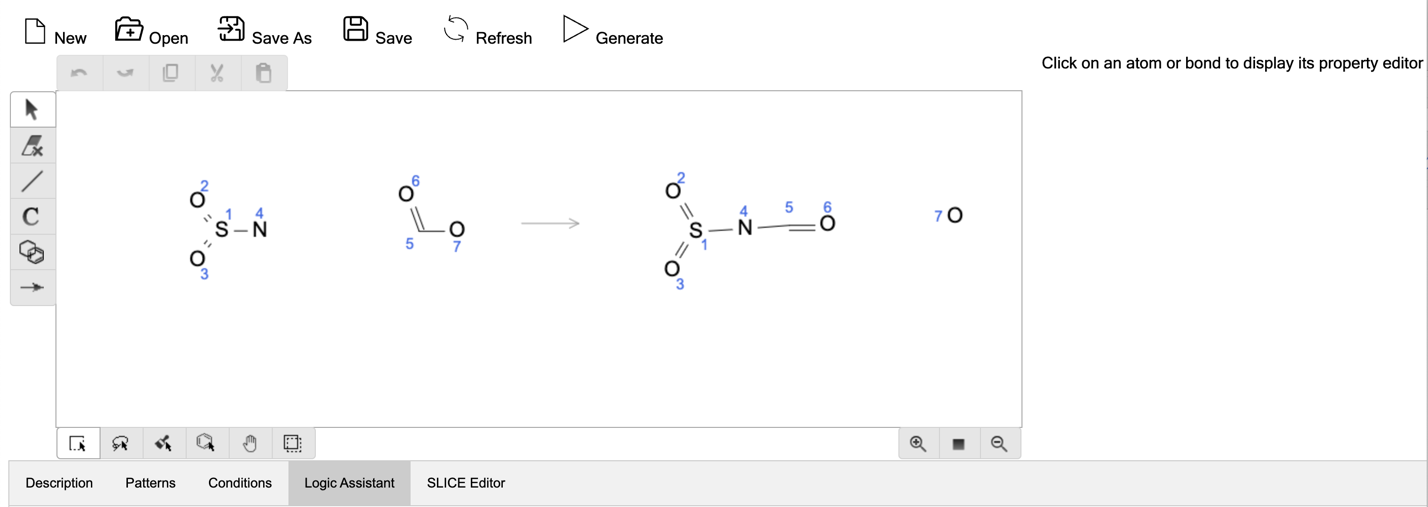


**Reactant 1**

**Reactant 2**

**Product 1**

**Product 2 as ghost**

Ghost molecules are artificial fragments that are not "real" products but are required to balance a reaction. For example, in transform 7009, a water molecule (H2​O) is defined as a ghost molecule because it helps balance the reaction without being a primary product.

**Step 2: Define Atom and Bond Properties**

In the SMARTS Editor Panel, configure the properties of each atom and bond in your reaction. This includes atom mapping and specific attributes like chemical element, negative charge, positive charge, connectivity, degree, hybridization number, total hydrogen count, unsaturation, periodic group, pi electron count, ring bond count, ring count, aliphatic hetero substituent count, hetero substituent count and valence. The software automatically generates the reaction's SMIRKS pattern, which you can view in the Patterns tab.

**SMARTS editor Panel**

Atom and bond properties

**Click on atom to open the SMARTS editor panel on the right**


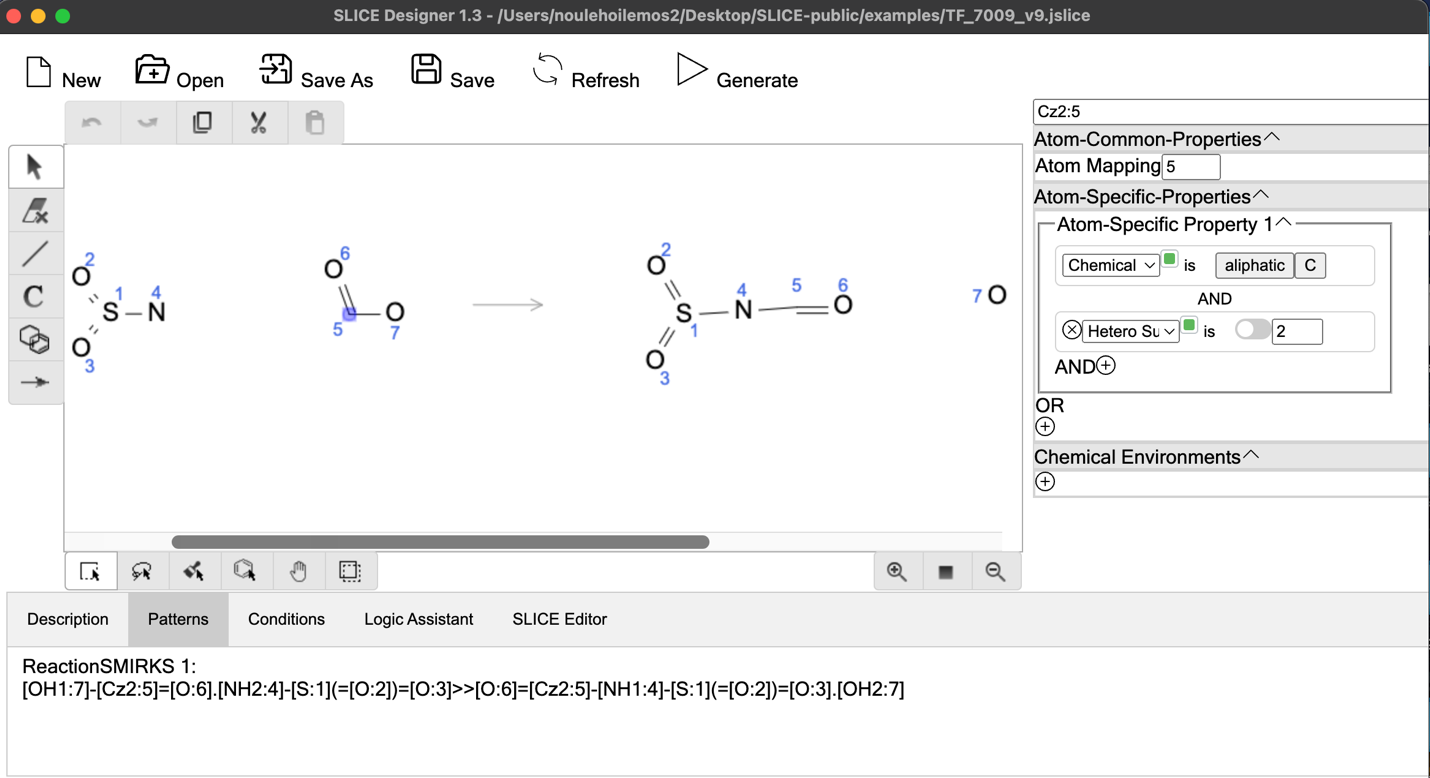


For example, you can define an atom as an aliphatic carbon, set its atom mapping to 5, and specify that it has two hetero substituents.

**Step 3. Write the Logic**

Select the Logic Assistant to define constraints for your reaction. Drag and drop blocks from the left-hand toolbox in the Window that opens.


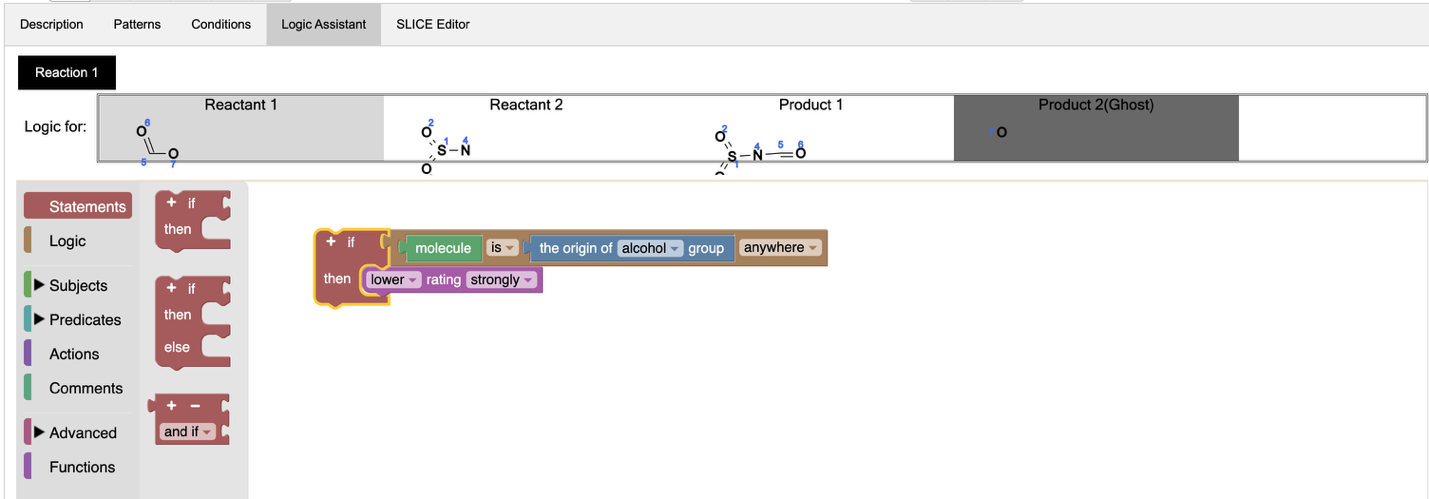


You can create simple rules using the block "IF".

Example of a simple constraint:

IF ANYWHERE THERE IS AN ALCOHOL THEN LOWER*RATING STRONGLY

a-) Select the block **if** in Statements (under toolbox)


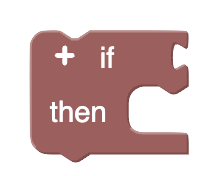


b-) Add the relation under **Logic** Toolbox by specifying where the logic should be applied. In this example the logic applies **ANYWHERE** on the molecule.


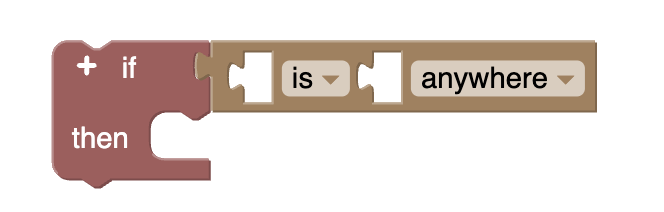


c-) Drag and drop the **subject** and **predicates.** In this example the subject is molecule and predicate is the alcohol group.


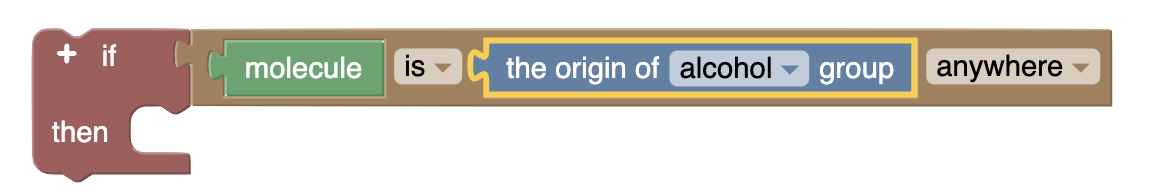


d-) Select **Action**


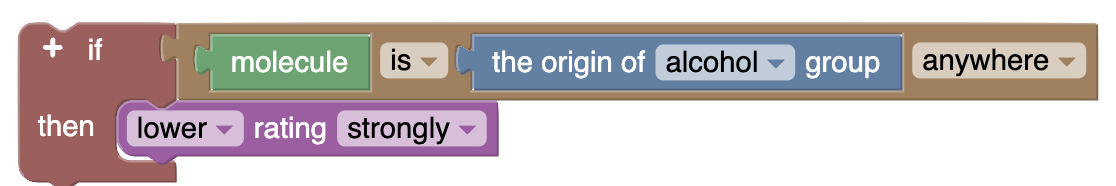


The block **and if /or if** can be used to group multiple constraints.


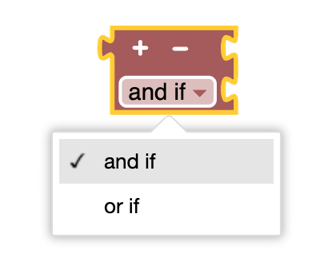


Here is a usage example to write these constraints in SLICE:

IF ANYWHERE THERE IS AN OXIME THEN LOWER*RATING STRONGLY

IF ANYWHERE THERE IS AN AMINE*1 OR: AMINE*2 THEN LOWER*RATING STRONGLY


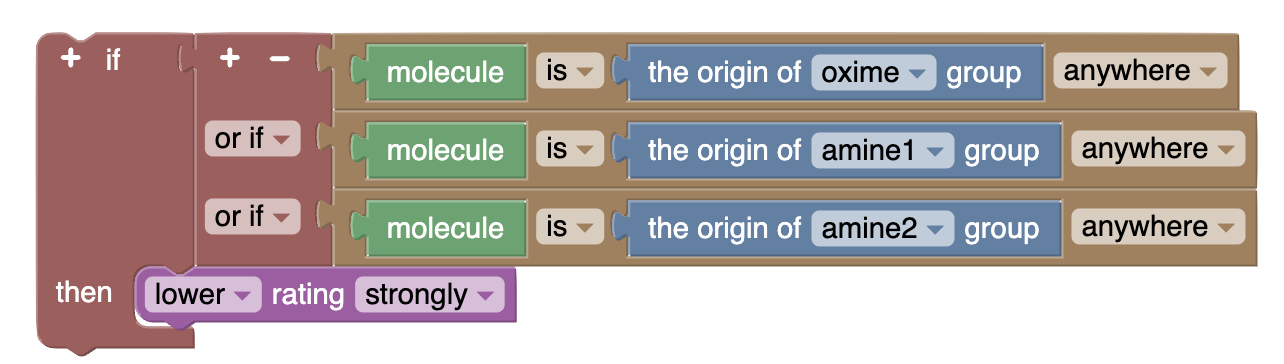


For more complex constraints such as this block:


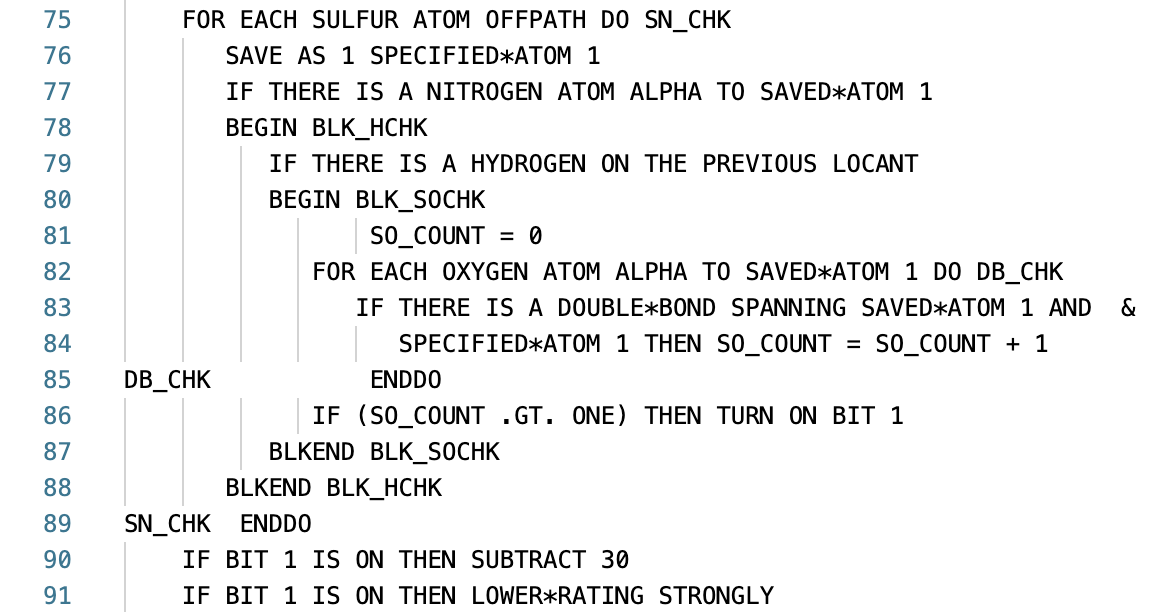


- Line 75 and 76 are interpreted with a loop **for** in SLICE Designer. The foreach option is in Advanced / Loops in the ToolBox


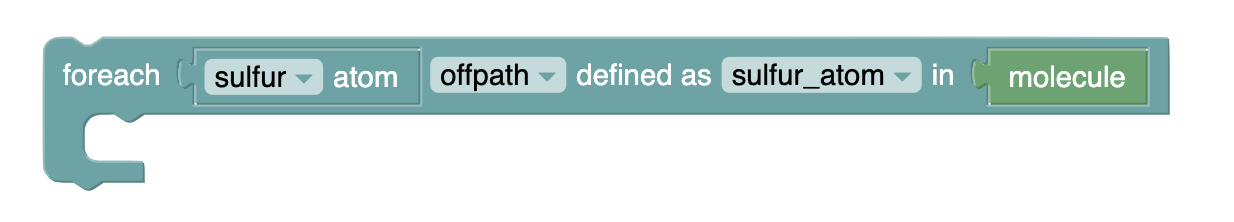


SPECIFIED*ATOM 1 is now the variable “sulfur_atom”.

- Line 77 checks all alpha atoms to SPECIFIED*ATOM 1 that are nitrogens.


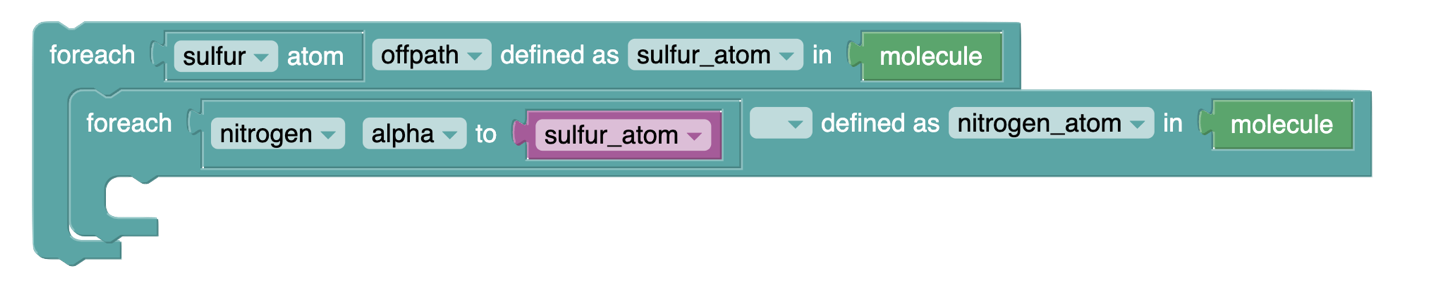


Nitrogen atoms are now referred as “nitrogen_atom”

- Line 79 checks if there is a hydrogen atom on “nitrogen_atom”


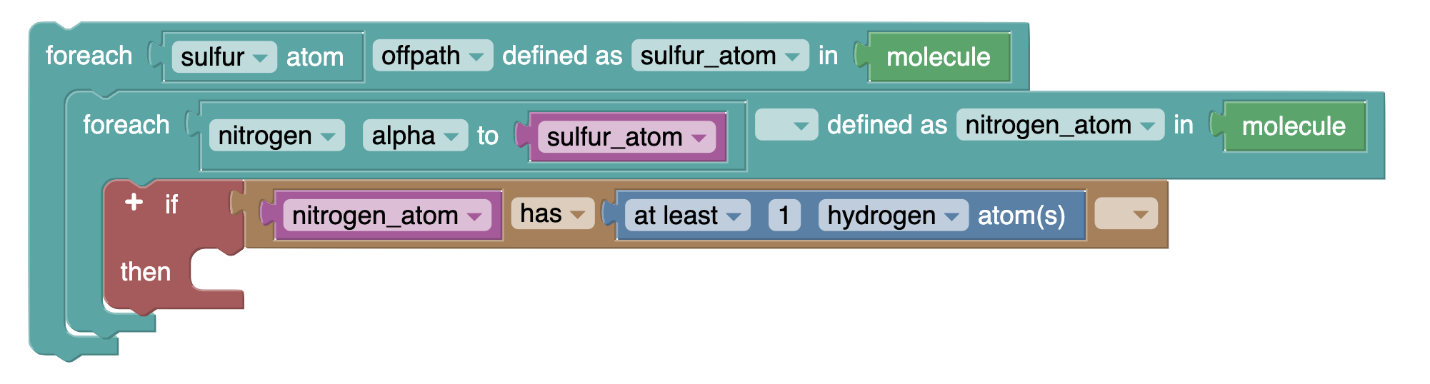


- For lines 81-84, we define a new variable so_count_local that will be incremented if there is a double bond between sulfur_atom and oxygen_atom


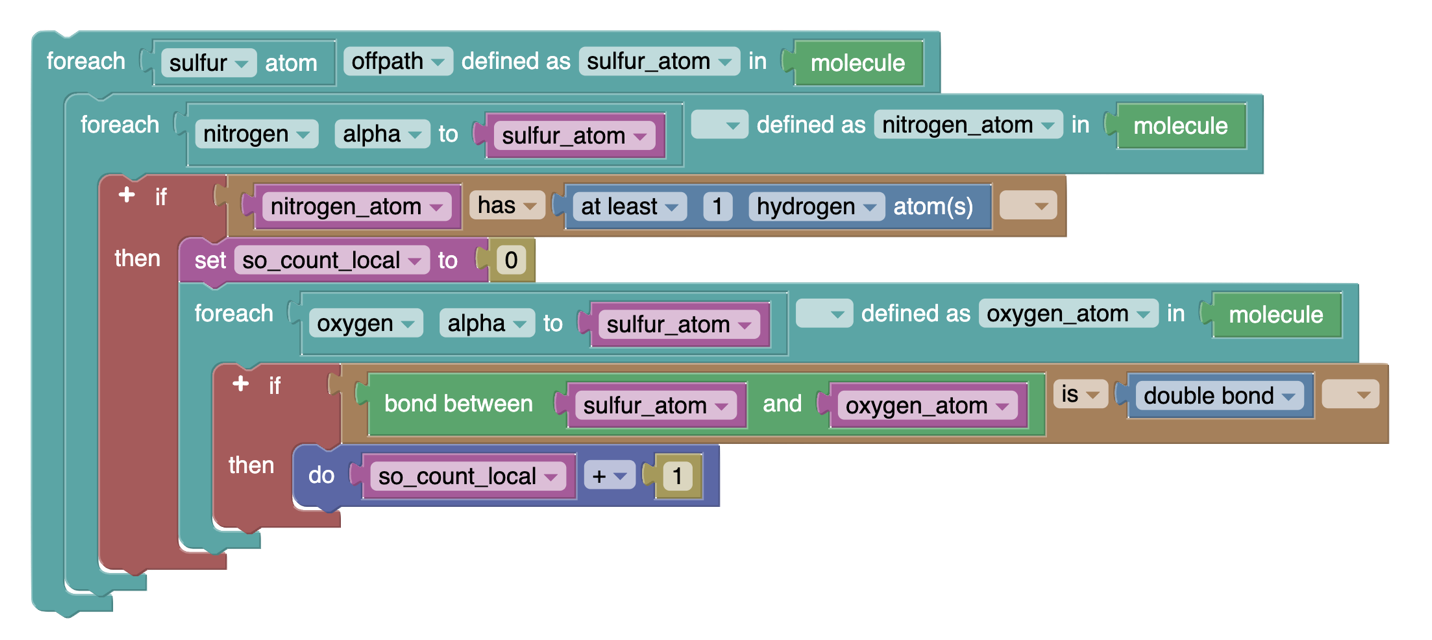


In the CHMTRN, BIT 1 represents a Boolean variable. In the SLICE implementation, the transform writer has chosen to use an integer variable, “so_count”.

Here is the complete logic for these complete constraints:


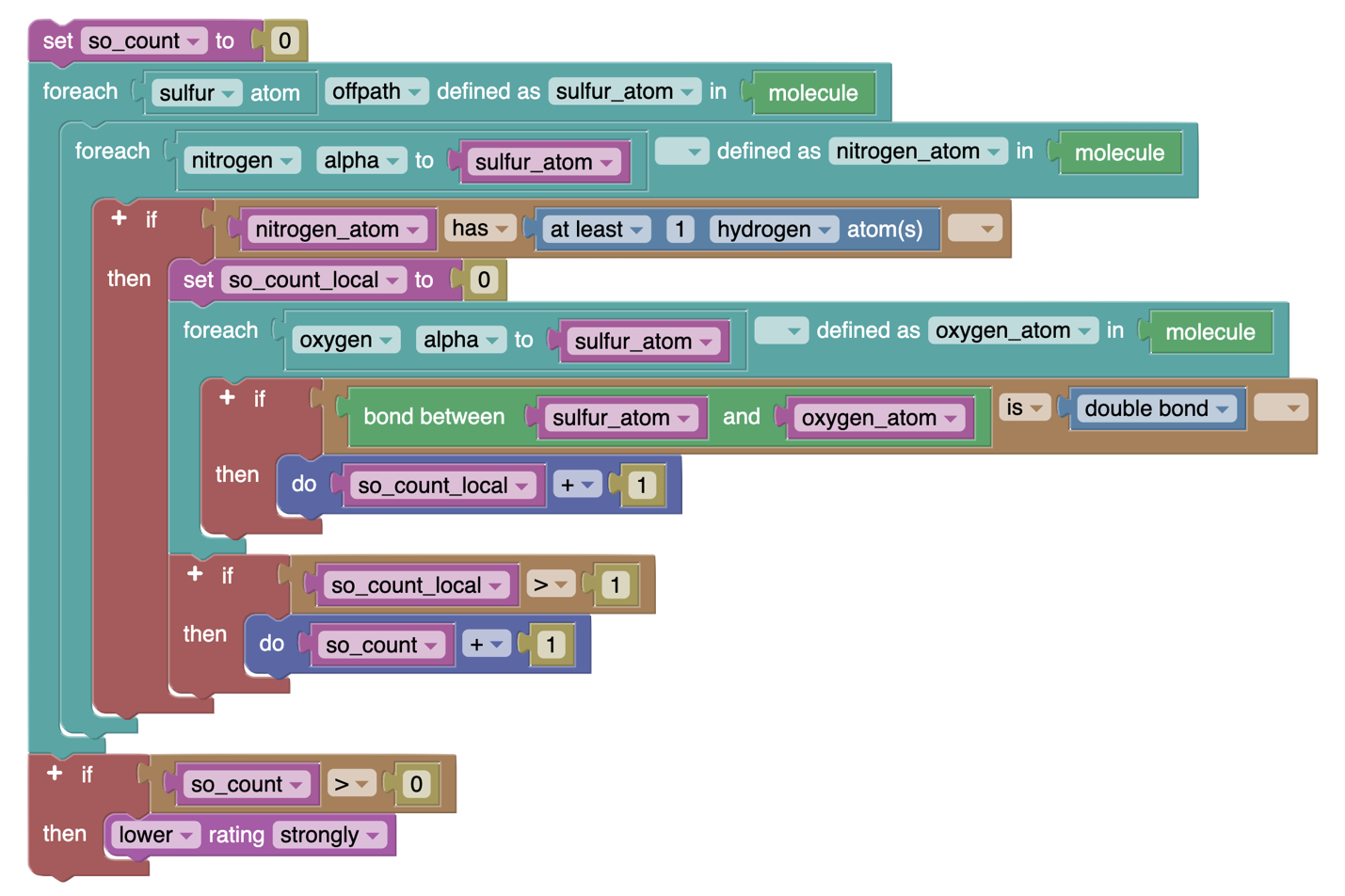


**Step 4**: **Generate products.**

After the logic is written and the file is saved, products can be generated using the button **Generate** on the top of the screen.


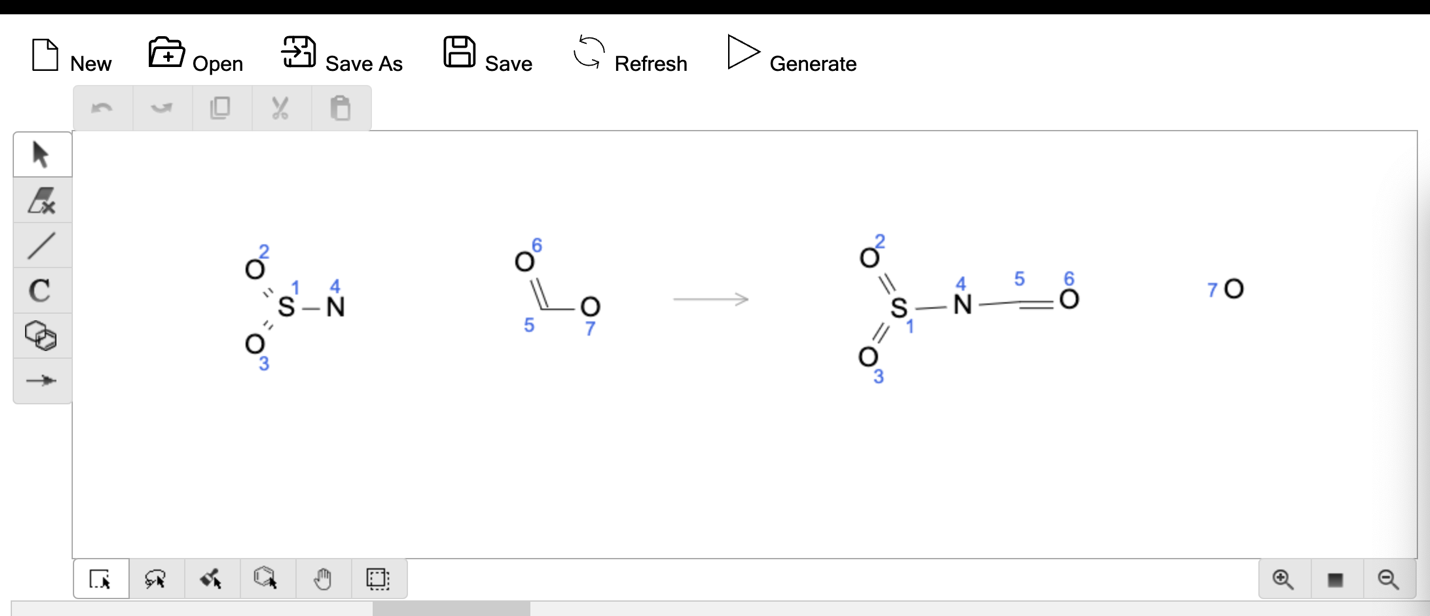


**Select the file with Building blocks**

**and the output format**

The input needed is a file with a column SMILES and column STRID (Enamine ID of the SMILES).

1. Click **"Display the number of compatible BBs"** to see how many building blocks are compatible with each reactant. The results are stored in text files, providing a detailed count for each reactant and a combined list.


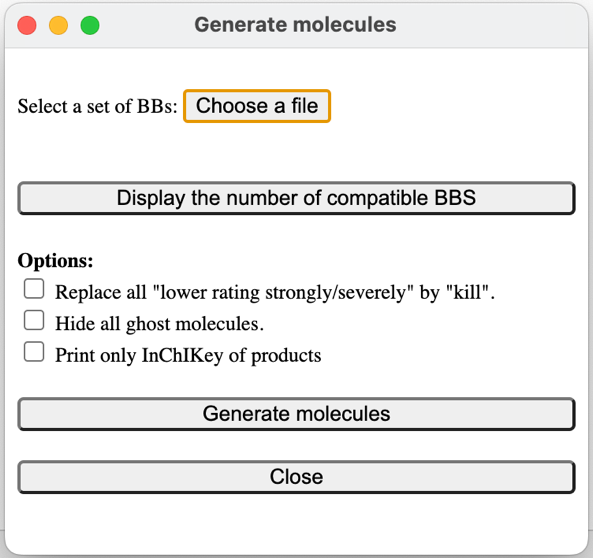


In this example, with TF 7009 and Enamine set of BBs of 284K these are the results:


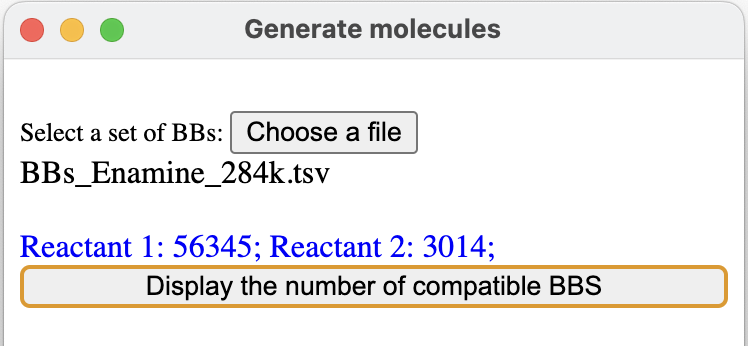


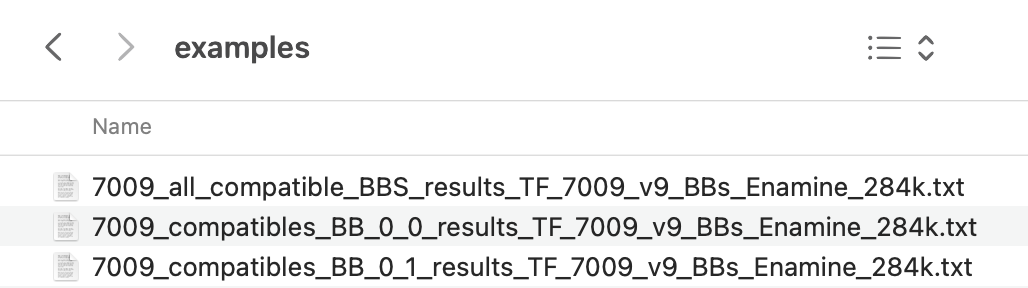


In this example,

**7009_compatibles_BB_0_0_results_TF_7009_v9_BBs_Enamine_284k.txt** : compatibles building blocks for reactant 1.

**7009_compatibles_BB_0_1_results_TF_7009_v9_BBs_Enamine_284k.txt** : compatibles building blocks for reactant 2.

**7009_all_compatible_BBS_results_TF_7009_v9_BBs_Enamine_284k.txt** : concatenation of compatibles BB for reactants 1 and 2.

1. Click **"Generate Molecules"** to produce the final output. By default, the output is an **.SDF** file containing all generated products. The ID of each product is a concatenation of the IDs of its two reactants.

You have three output options:

- **Hide all ghost molecules:** Excludes ghost molecules from the output file.
- **Print only InChIKey of products:** Generates a smaller output file containing only the InChIKey of each product, which is useful when dealing with a large number of products, as .SDF files can become very large.


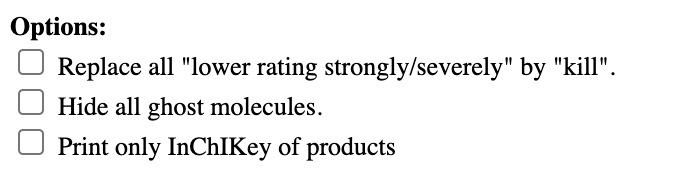

Supplement: Supplementary file 1 — Supplementary material 1. [file 13321_2025_1119_MOESM1_ESM.docx]
